# Relationship of serum homocysteine and vitamin D with positive, negative, and extrapyramidal symptoms in schizophrenia: a case–control study in Iran

**DOI:** 10.1186/s12888-022-04246-x

**Published:** 2022-11-04

**Authors:** Najmeh Shahini, Seyed Mohammad Mousavi Zade Jazayeri, Reza Jahanshahi, Abdurrahman Charkazi

**Affiliations:** 1 Golestan Research Center of Psychiatry (GRCP), Golestan University of Medical Sciences, Gorgan, Iran; 2 BSN, Student research committee, Golestan University of Medical Sciences, Gorgan, Iran; 3 Health Education and Promotion, Faculty of Health, Environmental Health Research Center, Golestan University of Medical Sciences, Gorgan, Iran

**Keywords:** Homocysteine, Vitamin D, Schizophrenia, Biomarker, Extrapyramidal, Iran

## Abstract

**Background:**

Schizophrenia is a devastating condition characterized by frequent recurrences, cognitive decline, and emotional and functional disabilities. This condition includes positive and negative symptoms and cognitive impairments resistant to drug treatment. According to studies, many biomarkers can affect this disorder. However, there is little information about vitamin D and homocysteine levels in patients with disease complications. We aimed to investigate this relationship in schizophrenia.

**Method:**

In this case–control study, 33 patients with schizophrenia and 33 healthy individuals were enrolled from Golestan, the north of Iran, in 2021. Blood samples were taken from all participants to assess vitamin D and homocysteine serum levels. In addition, schizophrenic patients completed the Positive And Negative Syndrome Scale (PANSS) and Simpson-Angus Extrapyramidal Side Effects Scale (SAS). Data analysis was performed at a significance level of 0.05 using SPSS 16 software.

**Results:**

Of the 66 participants, 66.7% had vitamin D deficiency, and 71.2% had normal homocysteine levels. However, the serum level of vitamin D was lower in schizophrenic patients than in controls (*p* = 0.035), and serum homocysteine levels were higher in the schizophrenic group than in controls (*p* < 0.001). Vitamin D levels in patients with schizophrenia were significantly correlated with the overall assessment of extrapyramidal symptoms (*r* = 0.35, *p* = 0.04). However, no significant relationship existed between vitamin D and homocysteine levels and PANSS results (*p* > 0.05).

**Conclusion:**

Serum levels of vitamin D and homocysteine were significantly lower and higher in schizophrenic patients than in the control group. Improvement of extrapyramidal symptoms in schizophrenic patients had a direct and significant relationship with serum vitamin D.

## Introduction

More than 50 million people worldwide have schizophrenia, the most severe and costly disease due to the prevalence of refractory symptoms [1]. Schizophrenia is a destructive disease characterized by frequent recurrences, cognitive decline, and emotional and functional disabilities. This disorder includes positive (hallucinations, delusions) and negative (emotional turmoil, apathy) symptoms and cognitive impairments highly resistant to drug treatment [2, 3]. Schizophrenia is a severe psychiatric disorder of unknown origin. Many studies have examined the biological process of the disease over the years [4]. Previous studies have assessed various schizophrenia components, including vitamin D, folate levels, vitamin B12, and homocysteine [5].

Homocysteine, a sulfur-containing amino acid, is involved in the methionine cycle, affecting brain development through multiple cellular pathways [6]. There is evidence that homocysteine levels are associated with psychiatric disorders such as Alzheimer’s disease [7], affective disorders, and schizophrenia [8–10]. Elevated plasma homocysteine may be a highly toxic metabolite for the brain and a risk factor for cardiovascular and other diseases, including heart attack, carotid stenosis, cerebral hemorrhage, dementia, bipolar disorder, depression, and Parkinson’s disease [5, 11]. Several studies have suggested an association between homocysteine and schizophrenia and reported elevated serum homocysteine levels in acute and chronic schizophrenia, which may play a role in its psychopathology [12, 13].

On the other hand, vitamin D is a unique neurohormone that may play an essential role in the onset of psychiatric diseases [14]. However, the association of vitamin D with psychiatric disorders is not well understood. Vitamin D receptors are widely expressed in the human brain. Vitamin D regulates several pathways of neurotransmitter transmission, including serotonin, dopamine, glutamine, and norepinephrine [15]. In this case, it is not far from the mind that low vitamin D levels are associated with various mental illnesses such as schizophrenia, depression, attention deficit hyperactivity disorder, and autism spectrum disorder [16, 17].

Although much is known about homocysteine and vitamin D levels in patients with schizophrenia, a few studies have assessed their relationship with schizophrenia, especially among Iranians [18, 19].

We hypothesized that vitamin D levels are lower and homocysteine levels are higher in patients with schizophrenia than in the control group. This study aimed to 1) investigate the serum levels of homocysteine and vitamin D in a north Iranian sample of schizophrenia patients compared to healthy controls and 2) assess the relationship between serum levels of vitamin D and homocysteine and the severity of extrapyramidal side effects and positive/negative syndrome in schizophrenia patients.

## Method

### Study design

A case–control study was conducted at Panj-Azar hospital in Gorgan, north of Iran, from May 2021 to July 2021.

### Participants

Thirty-three participants with no known mental illnesses were recruited as the control group and 33 schizophrenic patients as the case group, matched for age and sex.

### Eligibility criteria

The inclusion criteria included a) age over 18 years, b) schizophrenia confirmed by a psychiatrist at Panj-Azar hospital, Gorgan, Iran, using semi-structural interviews based on DSM-V criteria, c) no other physical or psychiatric illnesses in patients diagnosed with schizophrenia, d) undergoing treatment with atypical antipsychotics, and e) no psychiatric symptoms such as depression or history of psychiatric illness in the family in the control group. The exclusion criteria were a) another psychiatric illness, b) substance use disorder, c) metabolic disorder affecting serum vitamin D levels, and d) vitamin supplements affecting serum homocysteine and vitamin D.

### Sample size

The sample size was calculated using Eq. 1, considering the power of 80% and type 1 error of 5%.

1 $$n=\frac{\left({s}_{1}^{2}+{s}_{2}^{2}\right)*{({z}_{1-\frac{\alpha }{2}}+{z}_{1-\beta })}^{2}}{{d}^{2}}$$

According to Yanchi et al. [2], the total sample size was estimated at 66 people. Due to the outbreak of the coronavirus pandemic, the number of referrals to the hospital’s psychiatric department was meager, and we had to use a convenience sampling method.

### Psychiatric evaluations

Demographics and clinical information of the participants were collected through a checklist, including gender, age, BMI, education, and marital status. All information was obtained through face-to-face interviews. All diagnostic symptom evaluations were performed by the same psychiatrist using the Positive and Negative Syndrome Scale for Schizophrenia (PANSS) [20] and Simpson-Angus Extrapyramidal Side Effects Scale (SAS) [21]. In short, the PANSS measures the severity of symptoms in patients with schizophrenia and evaluates the positive and negative symptoms of psychosis. The items were divided into five areas: Positive factor (P1, P3, P5, G9), Negative factor (N1, N2, N3, N4, N6, G7), Disorganized/concrete factor (P2, N5, G11), Excited factor (P4, P7, G9, G14), and Depressed factor (G2, G3, G6) by Wallwork et al. based on the original version [22]. The items scored 1 (asymptomatic) to 7 (significantly symptomatic). This questionnaire is valid and reliable in Iran [23]. The SAS assesses the severity of extrapyramidal side effects in patients with schizophrenia. The rater asks the patient to perform 10 tasks and rates responses on a scale of 0–4 (normal to severe). Its validity and reliability were measured and confirmed in Iran [24].

### Blood collection

A venous blood sample (3 cc) was collected from each patient in a tube containing silicate gel to test serum levels of homocysteine and vitamin D. The samples were then stored at -20 °C. After the complete collection of samples, homocysteine and then vitamin D were evaluated in a thigh. The participant’s blood was collected immediately after they answered the questions.

### Homocysteine and vitamin D measurement

The ARA TECH kit measured serum vitamin D levels, and the BIOREXFARS kit measured serum homocysteine levels. According to ARA TECH’s vitamin D level standards, the participants were divided into four sub-groups: less than 10 as deficient, 10–30 as insufficient, 30–100 as normal, and more than 100 as toxic. According to the given criteria, the homocysteine levels of the participants in the BIOREXFARS kit were divided into normal (less than 13) and toxic (more than 13).

### Statistical analysis

Data are expressed as means and standard deviations. The Mann–Whitney test compared the two groups, and the Kruskal–Wallis test compared the means of more than two groups. Spearman’s correlation test examined the relationship between quantitative variables. Statistical tests were performed using SPSS version 16 software. The significance level was set at a *p*-value < 0.05. No data was missed because the interviewer collected the information.

### Ethical consideration

This study was conducted after obtaining ethical approval (IR.GOUMS.REC.1400.010) from the Golestan University of Medical Sciences.

## Results

**Fig. 1:**
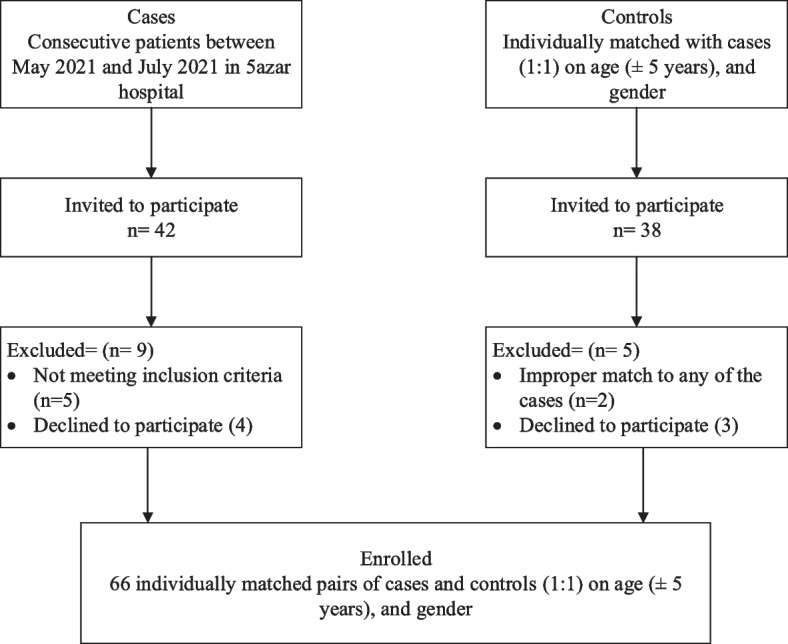
Flow chart describing the enrollment of schizophrenic cases and healthy controls

During the data collection period, 40 patients with schizophrenia were identified. Three patients were excluded due to a lack of informed consent to participate, and four were excluded due to the lack of required criteria. Overall, 33 patients completed the study (Fig. 1).

The demographic characteristics of the participants are shown in Table 1. The case and control groups were closely matched for potential confounders, including age and gender. However, marital status and BMI differed significantly between the two groups (*p* =  < 0.001 and 0.003, respectively).

**Table 1:** Demographic and laboratory characteristics of participants

Variables	Schizophrenia (*n* = 33)	Control (*n* = 33)	*P*-value
Gender			
Male	25 (75.8%)	25 (75.8%)	0.99
Female	8 (24.2%)	8 (24.2%)	
Marital status			
Single	19 (57.6%)	6 (18.2%)	0.001
Married	14 (42.4%)	27 (81.8%)	
Educational level			
High school	17 (51.5%)	11 (33.3%)	0.056
Diploma	13 (39.4%)	13 (39.4%)	
University	3 (9.1%)	9 (27.3%)	
BMI (kg/m^2^)			
Normal (18.5–23.9)	20 (60.6%)	8 (24.2%)	0.003
Overweight (24.0–27.9)	10 (30.3%)	17 (51.5%)	
Obesity (≥ 28.0)	8 (24.2%)	8 (24.2%)	
Age (year)	40 (34–47)	39 (28.5–49.5)	0.77
Vitamin D (µg/dl)	5.3 (1.75–9.65)	9.1 (4.5–19.5)	0.035
< 10	25 (75.8%)	19 (57.6%)	0.107
10.1–30	7 (21.2%)	11 (33.3%)	
30.1–100	1 (3%)	3 (9.1%)	
Homocysteine (µg/dl)	12.4 (10.95–16.2)	7.2 (5.5–9.6)	< 0.001
13 ≥	17 (51.5%)	30 (90.9%)	< 0.001
13 <	16 (48.5%)	3 (9.1%)	

Values are presented as n (%), median and interquartile range (1_st_ IQR- 3_rd_ IQR)

*BMI* Body mass index

Significant at *p* < 0.05 compared to the control group

### Comparison of serum levels of vitamin D and homocysteine between schizophrenic patients and healthy controls

Of all the participants, 66.7% (*n* = 44) had deficient levels, 27.3% (*n* = 18) had insufficient levels, and 6.1% (*n* = 4) had normal levels of vitamin D. None had vitamin D toxicity. The difference between the two groups was statistically significant, and the serum level of vitamin D was lower in schizophrenic patients than in controls (*p* = 0.035) (Table 1).

Additionally, 47 (71.2%) participants had normal homocysteine levels, and 19 (28.8%) had toxic levels. The difference between the two groups was statistically significant, and serum homocysteine levels were higher in the schizophrenic group than in the control group (*p* < 0.001) (Table 1).

### Correlation between serum levels of vitamin D and homocysteine

The Spearman correlation test revealed an inverse and significant relationship between vitamin D serum levels and homocysteine (*r* = -0.0258, *p* < 0.05).

### Results of SAS and PANSS questionnaires

Data obtained from questionnaires completed by patients with schizophrenia are presented in Table 2. There was a significant association between the positive factor of PANSS and SAS (*r* = 0.347, *p* = 0.04). The data showed no significant relationship between vitamin D and homocysteine levels and the PANSS factors (*p* > 0.05). As shown in Table 2, only vitamin D levels in patients with schizophrenia were significantly correlated with the overall assessment of extrapyramidal symptoms (*r* = 0.35, *p* = 0.04). None of the demographic characteristics had a significant relationship with the PNASS and SAS factors (*p* > 0.05).

**Table 2:** Relationship between the results of the questionnaires and laboratory parameters in the group of schizophrenics

	Median ± IQR	Vitamin D		Homocysteine	
		r	*p*-value	r	*p*-value
SAS	3 ± 3.5	0.355	0.04	-0.174	0.33
P-PANSS	16 ± 4	0.0	1	-0.214	0.23
N-PANSS	16 ± 5.5	0.116	0.52	0.103	0.56
Disorganized/concrete	8 ± 2.5	-0.164	0.36	-0.122	0.49
E-PANSS	18 ± 4.5	-0.2	0.26	0.744	0.059
D-PANSS	9 ± 4	0.278	0.11	0.24	0.17

*SAS* Simpson-Angus Extrapyramidal Side Effects Scale, *P-PANSS* Positive PANSS, *N-PANSS* Negative PANSS, *E-PANSS* Excited factor, *D-PANSS* Depressed PANSS

## Discussion

Serum vitamin D and homocysteine levels were significantly lower and higher in schizophrenic patients than in the control group. Consistent with our findings, a meta-analysis reported that patients with schizophrenia had lower vitamin D levels than healthy subjects or other psychiatric patients. Furthermore, a higher incidence of schizophrenia occurred in people with lower vitamin D. Therefore, we can understand a relationship between vitamin D and schizophrenia, but the cause remains unclear [25]. In agreement with our results, many studies reported that 55% to 65% of schizophrenia patients had vitamin D deficiency; in other words, studies report lower vitamin D levels in people with schizophrenia than in healthy people [26–28]. In contrast, studies showed that vitamin D serum levels were low but not statistically significant in patients with schizophrenia compared to healthy individuals [29, 30]. This difference in results may be due to the non-clinical study population or different ethnic groups.

As mentioned, serum homocysteine levels were significantly higher in patients with schizophrenia than in healthy people. In line with our results, a study of 760 schizophrenic patients reported that schizophrenic patients with depressive symptoms had elevated homocysteine levels compared to those without these symptoms [31]. Elevated homocysteine levels have been widely reported in schizophrenia and major depressive disorder [32–34]. The cause of elevated plasma homocysteine in schizophrenic patients is unclear, but studies have shown that malnutrition, coffee drinking, smoking, and inactivity can lead to elevated homocysteine levels [35]. Inadequate intake of vitamins B2, B6, and B12 is also a cause of hyperhomocysteinemia. Lack of vitamins B12 and B2 interferes with homocysteine remethylation, and vitamin B6 deficiency slows down absorption [36]. Due to the complications associated with hyperhomocysteinemia mentioned in the studies and the significantly high homocysteine levels in schizophrenic patients, action must be taken to lower homocysteine levels.

Our findings showed no association between vitamin D and homocysteine and the severity of positive and negative symptoms. After eight weeks of follow-up, there was no difference between vitamin D and placebo in terms of mental illness severity or metabolic status [3]. Bruins et al. reported the same results [37]. However, Rizki et al. [38], like Song et al. [19], found a significant correlation between positive and negative PANSS scores and homocysteine levels. This difference in results may be due to the low sample size of our study. Our study also suggested a link between vitamin D and extrapyramidal side effects. According to a literature review, no study had focused on the relationship between vitamin D levels and extrapyramidal symptoms, making it difficult to discuss this topic. However, several studies have found that high vitamins B12, C, and E can help reduce extrapyramidal symptoms [39, 40].

Our study’s interesting finding was the relationship between the PANSS positive factor and the SAS score. Other researchers have found a link between PANSS negative symptoms and SAS score [41, 42]. There may be two causes for this inconsistency in results: the modification made in the negative and positive sections’ questions of the PANSS in our study and the other data on medicines used by patients evaluated in prior research, whose information was not obtained in the current study.

This study was conducted for the first time in northern Iran. Vitamin D levels have been reported to be low in healthy people in the region due to the lack of sun exposure, which was even lower in patients with schizophrenia. One of the study’s strengths is its methodology, based on the STROBE checklist, and the appropriate control of potential confounders. One of the limitations of this study is the small sample size and failure to collect treatment information, such as duration and medication type. Other limitations were that it did not consider the possible relationship between drug types and doses and vitamin D and homocysteine serum levels. The link between vitamin D levels and extrapyramidal side effects was demonstrated in this study for the first time; however, a firm conclusion regarding this issue cannot be reached owing to a lack of data about medicines taken by the patients. It is recommended that researchers investigate the association between these two factors, taking into account a larger sample and a more extensive inquiry into the type of medicines used and the duration of treatment. Finally, the PANSS was used to assess negative symptoms, which was the study’s final limitation. According to a 2021 research by Galderisi et al. [43], the PANSS items cannot adequately explore negative symptoms and should be replaced with alternative questionnaires. However, based on the literature research, we attempted to address this issue by removing the items that generated assessment inaccuracies [22, 43, 44].

## Conclusion

Serum homocysteine levels were significantly higher in schizophrenic patients than in the general population. Also, serum vitamin D levels were significantly lower in schizophrenic patients than in the general population. Improving extrapyramidal symptoms in schizophrenic patients had a direct and significant relationship with serum vitamin D. We recommend nutritional supplements to improve serum vitamin D and homocysteine levels and monitor them in patients’ sera.

## Data Availability

Not applicable.

## Abbreviations

PANSS: Positive and Negative Syndrome Scale for Schizophrenia
SAS: Simpson-Angus Extrapyramidal Side Effects Scale
GP: General Psychopathology
cc: Cubic centimeter
°C: The degree Celsius
